# Management of Liver Oligometastatic Esophageal Cancer: Overview and Critical Analysis of the Different Loco-Regional Treatments

**DOI:** 10.3390/cancers12010020

**Published:** 2019-12-19

**Authors:** Fabio Procopio, Salvatore Marano, Damiano Gentile, Anna Da Roit, Silvia Basato, Pietro Riva, Ferdinando De Vita, Guido Torzilli, Carlo Castoro

**Affiliations:** 1 Division of Hepatobiliary and General Surgery, Department of Surgery, Humanitas University, Humanitas Clinical and Research Center-IRCCS, Via Manzoni 56, Rozzano, 20089 Milan, Italy; fabio.procopio@humanitas.it (F.P.); damiano.gentile@humanitas.it (D.G.); guido.torzilli@hunimed.eu (G.T.); 2 Division of Upper Gastro-Intestinal Surgery, Department of Surgery, Humanitas University, Humanitas Clinical and Research Center-IRCCS, Via Manzoni 56, Rozzano, 20089 Milan, Italy; salvatore.marano@humanitas.it (S.M.); anna.da_roit@humanitas.it (A.D.R.); silvia.basato@humanitas.it (S.B.); pietro.riva@hunimed.eu (P.R.); 3 Division of Medical Oncology, Department of Precision Medicine, School of Medicine, University of Study of Campania “Luigi Vanvitelli”, Via Pansini 5, 80131 Naples, Italy; ferdinando.devita@unicampania.it

**Keywords:** esophageal cancer, liver oligometastatic esophageal cancer, noncolorectal nonneuroendocrine liver metastasis

## Abstract

Esophageal cancer (EC) is an aggressive disease that is associated with a poor prognosis. Since metastastic EC is usually considered suitable only for palliative therapy with an estimated 5-year overall survival (OS) less than 5%, the optimal management of patients with liver oligometastatic EC (LOEC) is still undefined. The aim of this review is to provide an overview of the different treatment options for LOEC. A literature search was conducted using PubMed, Embase, and Cochrane to identify articles evaluating different treatment strategies for LOEC. Among 828 records that were identified, 20 articles met the inclusion criteria. These studies included patients who have undergone any type of surgical procedure and/or loco-regional therapy. Liver resection resulted in the best survival for patients with low tumor burden (3 lesions): 5-year OS 30–50% versus 8–12% after only chemotherapy (CHT). The 5-year OS of loco-regional therapies was 23% with a local recurrence risk ranging 0–8% for small lesions (2 to 3 cm). An aggressive multidisciplinary approach for LOEC patients may improve survival. Surgery seems to be the treatment of choice for resectable LOEC. If unfeasible, loco-regional therapies may be considered. In order to better select these patients and offer a chance of cure, prospective trials and a definition of treatment protocols are needed.

## 1. Introduction

Over the last twenty years, the incidence of esophageal cancer (EC) has rapidly increased with approximately half a million new cases each year [1]. EC still represents an aggressive disease that is associated with a poor prognosis [2,3]. At present, a surgical approach combined with chemotherapy (CHT)/chemo-radiotherapy represents the preferred treatment [4,5,6]. The five-year survival rate for patients undergoing surgical resection is now approaching 50% [7,8]. Patients with metastastic disease are usually considered suitable only for palliative therapy with a 5-year estimated survival less than 5% [2,9]. The liver is one of the most common sites of metastases, occurring in 35–40% of patients at the time of EC diagnosis [1,10] and it is the first recurrence site in 6–25% cases after esophagectomy [11,12,13]. The optimal management of patients with liver oligometastatic EC (OEC) is still a question of debate. Surgical resection for liver metastases is usually not pursued due to oncological reasons. Alternative therapeutic options generally include CHT and loco-regional therapies. More recently, small trials have reported that patients with low hepatic tumor burden have a favorable prognosis after CHT followed by liver resection [9,14,15,16]. Indeed, the prognosis of patients with non-surgically treated liver OEC still remains largely unknown. Although primary EC is frequently sensitive to multimodal treatment, metastatic EC generally does not respond well to CHT, therefore medical treatment alone does not offer the possibility of a cure. With the current CHT regimen, a complete response is rare, the duration of the response is short, and survival is usually 6 to 8 months [17]. Therefore, in selected patients with liver OEC that is not refractory to standard medical therapies, a multimodal approach including liver resection and/or other local therapies might be considered. An enlarged multidisciplinary approach that considers all these treatment options may optimize the management of the disease and maximize the pool of treated patients. The aim of this review is to provide an overview of different treatment options for OEC. The most relevant evidences in the literature are considered and the key issue is discussed.

## 2. Methods

### 2.1. Search Strategy

A systematic search of the literature was performed. The search aimed to identify all published researches that evaluated different treatment strategies for liver OEC. Research databases included PubMed, Embase, and Cochrane. The following terms were used for the search strategy: “liver oligometastasis”, “non-colorectal non-neuroendocrine liver metastasis”, “liver resection”, “hepatic resection”, “hepatectomy”, “loco-regional treatment” in combination with “esophageal cancer” or “esophagectomy”. Either free-text and medical subject heading (MeSH) searches were used for keywords. The search was further broadened by extensive cross-checking of all the references in the retrieved articles fulfilling the inclusion criteria in order to identify eventual additional non indexed literature.

### 2.2. Eligibility Criteria

Two reviewers (FP and DG) independently reviewed all of the abstracts to identify studies that fulfilled the predetermined eligibility criteria. We considered all the studies that included patients who had undergone any type of surgical procedure and/or loco-regional therapy for liver OEC. Studies written in any other language from English were excluded.

### 2.3. Definition

Although we lack a precise and consistent definition, the oligometastatic disease is generally considered a relatively favorable clinical state, with more indolent biology, a limited number of disease sites, and with the potential for prolonged periods of disease control [18]. Even though the oligometastic gastric cancer is defined as a tumor burden less than 5 nodules [19,20], this is not the case with EC. Thus, we believe that definition criteria are mandatory in order to establish the right treatment. Based on our literature review, the definition seems merely based on personal opinion. However, the main factor in determining the right definition seems to include up to 3 lesions in the disease that are radically treatable by surgery and/or loco-regional therapies [21].

## 3. Results

### 3.1. Study Collection

**Figure 1:**
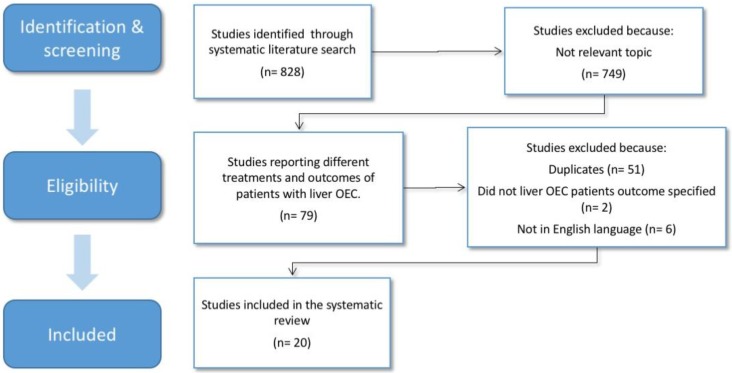
Diagram with a selection of studies for the review.

A total of 828 articles were identified after the primary literature search (Figure 1). After title and abstract review, 79 articles met the eligibility criteria for the full-text review. Following the full-text review, a total of 20 articles were included in the final analysis: 12 regarding the surgical treatment, 3 regarding ablative, and 5 regarding radiation therapy.

### 3.2. Surgery

At present, there are no available randomized trials comparing palliative and multimodal approaches (CHT versus CHT + surgery/ablation therapy) for liver OEC or comparative studies with propensity score match and meta-analyses. Most available data are based on few case reports or anecdotal information from Eastern centers and are consequently not suitable for formulating robust recommendations [9,22]. Although a general consensus has not been reached, small trials reported that patients with a low hepatic tumor burden (less than three lesions) have a favorable prognosis after liver resection irrespective of metachronous or synchronous disease.

We found some studies regarding synchronous disease [14,15,23,24,25]. Van Daele et al. [14] reported an overall survival (OS) and disease-free survival (DFS) of 50% and 33% at a median follow-up of 22 months after surgery, respectively. One third of the patients who were analyzed in the study had metastasectomy of 1 to 2 liver metastases during the same EC surgery. These findings are closely mirrored by Gandy et al. [23], with a 60% 5-year OS after liver resection in a cohort of 48 patients, 2 of which were affected by liver OEC. In a retrospective study including 96 patients, Wang et al. [15] reported that a small subset of 14 patients who were treated with a multimodal approach including liver resection showed a more favorable prognosis compared to patients who did not undergo surgery. Similarly, Carmona-Bayonas et al. [24] reported that 92 patients who underwent surgery for EC metastasis had a higher survival rate than patients who did not undergo metastasectomy (30% versus 8%). The most common surgeries were represented by peritoneal (29%), hepatic (24%), and distant lymph nodes (11%) resection. Concerning patients with liver metastases, 1 or 2 lesions were resected in 82% of the patients and 92% received CHT prior to surgery. In this subset of patients, the median OS was 17 months while 1-year and 3-year DFS were 42% and 35%, respectively. Slotta et al. [25] reported a mean survival of 12.5 months in patients treated by metastasectomy, underlining the striking impact of hepatic surgery in the prognosis of liver OEC disease.

We found similar results for metachronous disease [9,16,23,26,27]. At present, there is no guideline indication concerning the treatment of metachronous distant metastases of esophageal cancer. The decision to resect distant metastases is mostly a personalized treatment recommended by tumor boards for patients in good general condition with a long tumor-free survival. Adam et al. [26] reviewed 1452 patients who were resected for non-colorectal liver metastasis (CLM) and reported a 3-year OS of 32% in 20 (1.4%) patients who underwent the liver resection for hepatic OEC. In a recent retrospective study, Hiyoshi et al. [16] evaluated the clinical significance of surgery for recurrence after radical esophagectomy. Among 100 patients who underwent different treatments for OEC, 22 lesions in 14 patients were surgically treated—among them, one patient had metastasectomy for solitary liver metastasis. Overall, the surgical group showed a more favorable OS after both esophagectomy and initial recurrence compared to the non-surgical patients (*p* < 0.006 for both). Among patients with liver metastasis, the survivals were 35 and 23 months, respectively. Similarly, Liu et al. [27] showed the positive impact of surgery on survival for solitary liver metastasis compared to other loco-regional therapies. Moreover, the authors showed that a longer disease free interval, lasting more than 12 months from esophagectomy, may potentially be considered as a positive prognostic factor for the selection of patients for liver resection. Similarly, in their preliminary study, Huddy et al. [9] reported that a good response to CHT associated with the operability of the liver disease represents predictors of biologically favorable disease and therefore, potential selection criteria for a curative liver resection (Table 1).

### 3.3. Ablative Therapy

Over the last decade, radiofrequency ablation (RFA) has well established its place in the treatment algorithm of unresectable liver tumors [29]. In small lesions, RFA is considered the standard technique, with a complete response rate approaching 97% and 5-year survival rates up to 51% [30,31,32,33,34,35]. Several trials compared RFA with resection, with conflicting evidences reported. Some studies [36,37,38,39,40,41] did not show differences between resection and RFA in terms of overall and local tumor progression-free survival. However, these data are not conclusive because of methodology limitations. Large retrospective series [30,41,42], propensity score analyses [43,44], and meta-analyses [45,46] demonstrated the superiority of surgery over RFA in terms of local disease control, even for lesions of 2 to 3 cm in diameter.

Recently, several reports have demonstrated the beneficial local control effect of RFA in liver metastasis from gastric cancer (LMGC). Its oncological adequacy seems to be better demonstrated for small lesions (2 to 3 cm in diameter), similarly to CLM. However, the role of RFA in LMGC is not clearly defined, while data on the use of ablation for liver OEC are very scarce. However, these reports featured a very small and heterogeneous patient population.

Indeed, the literature search identified few potentially relevant publications [27,47,48,49]. Berber et al. [46] reported that cumulative local tumor control with RFA in non-CLM was inferior compared to other diseases such as hepatocellular carcinoma and neuroendocrine metastasis (82% versus 88–95%, *p* < 0.05). Contrarily, this inferiority was not evident when compared to CLM (82% versus 73%, *p* = n.s.). Indeed, local disease control after RFA was higher for lesion size less than 3 cm, regardless of the tumor type. Similarly, Littrup et al. [48] had comparable results, showing local recurrence (LR) rates of 9.4% for mean tumor sizes less than 3 cm: 6 of 99 patients had liver OEC. In conclusion, ablative therapy could be considered oncologically adequate for small liver OEC. The diatribe between ablation therapy and surgery is still open. More recently, Liu et al. [27] compared the effect of resection and RFA in patients with solitary liver metastasis from esophageal squamous cell carcinoma (SCC). Patients who were surgically treated had significantly higher survival than ablative treated patients (1- and 2-year OS 50% and 21% versus 31% and 7%; *p* < 0.005). Contrarily, Goering et al. [28] reported similar survival and LR risk between ablative and surgical treatment in a cohort of 42 patients (1 of them with liver OEC) with non-CLM (5-year OS 40% versus 37% *p* = 0.57; 3-year LR rate 24% versus 19%, *p* value not reported) (Table 2).

### 3.4. Radiotherapy

Regarding additional treatment strategies other than surgery and RFA, only a few more options have been reported in liver OEC, such as Stereotactic body radiation therapy (SBRT). SBRT delivers brief, high regimen, daily doses of focused external radiotherapy and is used for primary and secondary liver tumors with acceptable local disease control. At present, few reports have investigated the effectiveness of this treatment on patients with hepatic disease from non-colorectal malignancies. Of note, much of the data in support of radiotherapy have been extrapolated from heterogeneous groups of oligometastatic patients.

Although there have been no randomized data, retrospective studies and phase I/II trials have demonstrated that radiotherapy is well tolerated and associated with encouraging outcomes and local disease control. In their retrospective analysis, Blom et al. [50] analyzed the survival of 493 patients with recurrent EC after single or multimodality therapy; 425 patients had distant recurrence. The majority of these patients (*n* = 117) had liver oligometastases and 130 patients (26% of the total) with multiple sites oligometastases were treated with radiotherapy. The authors showed that curative treatment for recurrent disease is rarely possible, with a scarce survival benefit of 3–6 months. Tanaka et al. [51] treated 88 patients with distant oligometastases from esophageal SCC. Half of the patients (*n* = 40) were affected by liver metastases, followed by lung and bone lesions. Seven (8%) patients were treated with radiotherapy alone. The authors showed that the survival of patients treated with multimodality therapy including surgery was significantly better than that of patients who received single modality therapy (including radiotherapy alone) or best supportive care (*p* < 0.0001). Hong et al. [52] analyzed the efficacy and safety of SBRT in 89 patients with 1–4 liver metastases from different primary malignancies; 13% of these patients were affected by EC. The majority of these patients (*n* = 55) had a single metastasis with a median tumor size of 2.5 cm. Cumulative median survival was 18 months while 1- and 3-year local control rates were 72% and 61%, respectively. Rusthoven et al. [53] reported a multi-institution phase I/II study of SBRT for liver metastases. Patients were affected by 1 to 3 hepatic metastases with individual tumor diameter less than 6 cm. Forty-seven patients were enrolled and a total of 63 liver metastases were treated. Although patients with CLM were the best represented group (32% of patients), 6% of patients were treated for liver metastases from EC. The two-year local control rate was 92% (100% for tumors less than 3 cm). Fode et al. [54] analyzed 321 consecutive patients treated for 587 metastases with SBRT (68% were liver metastases). Patients were affected by 1–6 lesions with the size of the tumors ranging from 1 to 80 mm. Although CLM were the most represented lesions (63% versus 27% non-CLM), 1- and 2-year LR rates were 9% and 13%, respectively. Cancer type and size of the largest lesions were not related to LR risk (*p* = 0.25). This study suggests that SBRT also has an adequate efficacy in local disease control for non-CLM.

## 4. Discussion

The purpose of this review was to provide an overview of the different treatment options underlining the striking impact of a multimodal approach in the prognosis of patients with liver OEC. Although a general consensus about the management of these patients has not been reached, many of the before mentioned studies showed the potential benefit of an aggressive multimodal approach. As reported here, these results support the role of surgical treatment (both staged and simultaneous esophagus-liver resection) in those patients with low tumor burden (less than three lesions) [21]. A 5-year OS of 50% after liver resection versus 8–12% after CHT alone was reported. These results suggest that surgery has a significant impact on the prognosis of OEC patients. However, extrapolating from gains made in surgical treatment, it is assumed that such survival improvements are in part due to modern systemic CHT. Contemporary chemotherapeutics increased the number of patients who are able to undergo resection and improved recurrence-free survival [55,56]. Although it is manifestly too early to generalize and extrapolate robust conclusions, partial responses have been reported from all reported studies, suggesting the benefit of surgery when applied as a supplement to CHT. Given these considerations, patients with a low and resectable tumor burden should be approached with a combination of surgical and systemic therapy. These findings might contradict the dogma that liver resection should be precluded for patients with liver OEC. Although the appropriate timing of systemic therapy in resectable patients remains controversial and further studies are needed, it seems that the response to preoperative chemotherapy may be a major factor for surgical resection in patients with metastatic OEC [57]. Therefore, the appropriate timing of systemic therapy in resectable patients remains controversial and additional studies are needed. We are in line with the concept that disease chemo-responsiveness should be a sine qua non condition for a surgical approach.

In cases of significant surgical risk or small liver lesion, RFA might be considered. In general, ablative therapy remains a supplementary tool to resection and an option for patients with unresectable lesions in terms of comorbidities and cancer-specific factors. To date, no studies have been able to answer the question whether RFA could become an acceptable alternative to surgery for resectable liver OEC. Thus, more trials are needed to reach conclusive evidences, however surgical resection seems preferable to ablation for lesions more than 2 cm in diameter. Regarding patients with resectable CLM, current LR rates are still inadequate, except after RFA of small tumors (less than 2 cm in diameter) [31,32,33,34,35,36]. Indeed, the role of RFA in liver OEC still remains undetermined. Its limited application in such tumors is probably attributed to oncological outcome rather than technical procedure. From the point of view of tumor biology and a behavioral perspective, liver metastasis deriving from EC seems to be similar to LMGC. Therefore, there is a good basis in proposing a similar loco-regional therapeutic approach for patients with liver OEC.

All the authors agree that surgery and RFA are equivalent for very small lesions in terms of both local control rate and OS. However, the exception in this statement regards the anatomic position of the tumor or its contact with major vascular structures. For perivascular lesions, the therapeutic efficacy and safety of ablation is significantly reduced. Alternatively, for such lesions, microwave ablation (MWA) could be considered as the preferred technique [58]. However, when comparing RFA and MWA, patients in the MWA group seem to show a significantly lower LR rate (6% versus 20%) [59]. Therefore, surgical resection should represent the preferred option in such patients despite the small tumor size. The authors agree that lesions up to 2 cm that are neither sub-capsular nor peri-vascular represent the ideal target for ablation therapies.

Alternatively, in cases of predicted sub-optimal results from RFA, SBRT may be considered. Although evidence for SBRT on non-CLM is less established, the question remains open whether these results are related to a true clinical effect or simply to a selection of patients with a better prognosis. However, the present studies do not justify the exclusion of SBRT in oligometastatic patients. Further studies are needed to compare the efficacy of SBRT with those of surgical resection or RFA. The authors agree that lesions that are neither resectable nor suitable for ablative therapy should be considered for SBRT as an alternative treatment. At present, there are no trials comparing these treatments and the selection of the best treatment is patient-dependent.

Our review does have limitations, which includes its small sample of patients and the fact that it was performed mostly based on retrospective studies. It evaluates highly selected patients and applying these recommendations to patients must, therefore, be done carefully as the results are not necessarily generalizable to all patients. However, the review does have clinical merit as the results suggest that the liver OEC disease should not necessarily preclude a patient from surgical and local-regional treatments. Selection certainly is key and chemotherapy plays a dominant role, however some patients may benefit from aggressive treatment management. A meta-analysis would have been helpful to elucidate the “real” survival benefit of surgical and ablative approaches for the treatment of liver OEC. However, the meta-analysis was unfeasible due to the fact that in all the studies that we collected, there was no reference cohort of patients who received systemic chemotherapy (control group), only for those who did undergo surgery or local therapy (case group).

## 5. Conclusions

In conclusion, an aggressive multidisciplinary approach for patients with liver OEC seems to improve survival. However, the data is weak, yet there are anecdotal examples of good outcomes from surgery. If unfeasible, loco-regional therapies might be considered. However, in order to better select the patients and to offer a chance of cure or, at least, to optimize the prognosis, a clear definition of treatment protocols is needed. Given the risk of tumor recurrence, strict follow-up protocols as well as an iterative multidisciplinary approach are required. Moreover, there is a need for prospective trials in order to define the inclusion criteria, reliable predictors of better outcomes, and the optimal timing for CHT, surgery, and other local therapies.

## Figures and Tables

**Table 1:** Details of the results of the selected studies analyzing the oncological outcomes of patients with liver OEC treated with hepatic resection.

First Author	Year	Journal	Study Type	Timing of Metastasis	Pathology	No. Patients	No. Liver OEC Patients	Follow-Up (Month) *	Survival
Huddy et al. [9]	2015	*Dis. Esophagus.*	CR	Meta	Miscellaneous	4	4	37	OS 10–92 months DFS 5–92 months
Van Daele et al. [14]	2018	*Acta. Chir. Belg.*	RS	Sync	Miscellaneous	12	7	22	OS 50% DFS 33%
Wang et al. [15]	2016	*Am. J. Clin. Oncol.*	RS	Sync	Adeno	96	14	47	5-yr OS 50.5%
Hiyoshi et al. [16]	2015	*Ann. Surg. Oncol.*	RS	Meta	NR	113	1	23	OS 92 months DFS 11.4 months
Ichida et al. [21]	2013	*World J. Surg.*	RS	Meta	Adeno	138	5	5	OS 13 (months) *
Mudan et al. [22]	2010	*Hippokratia*	CR	Sync	Adeno	1	1	-	OS 29 months
Gandy et al. [23]	2017	*ANZ J. Surg.*	RS	Meta	Adeno	48	2	24	3-yr OS 83% 5-yr OS 60%
Carmona-Bayonas et al. [24]	2018	*Eur. J. Surg. Oncol.*	RS	Sync	Adeno	92	22	17	3-yr OS 30.6% 3-yr DFS 35%
Slotta et al. [25]	2014	*Int. J. Surg.*	RS	Sync	NR	101	2	18 **	OS 12.5 months
Adam et al. [26]	2006	*Ann. Surg.*	RS	Meta	NR	1452	20	16	3-yr OS 32%
Liu et al. [27]	2018	*ANZ J. Surg.*	RS	Meta	SCC	69	26	14	2-yr OS 21.2%
Goering et al. [28]	2002	*Am. J. Surg.*	RS	Meta	Adeno	42	1	45	5-yr OS 37%

OEC, oligometastatic esophageal cancer; RS, retrospective study; CR, case report; Sync, synchronous; Meta, metachronous; OS, overall survival; DFS, disease-free survival; NR, not reported; Adeno, adenocarcinoma; SCC, squamous cell carcinoma. * The values are expressed as the median. ** The value is expressed as the mean.

**Table 2:** Details of the results of the selected studies analyzing the oncological outcomes of patients with liver OEC treated with loco-regional therapies.

First Author	Year	Journal	Study Type	Treatment Type	Pathology	Patients	Liver OEC Tatients	Follow-up (month) *	Tumor Size (cm)	Survival
Berber et al. [47]	2008	*Ann. Surg. Oncol.*	RS	RFA	NR	335	NR	17	5	LR 22% OS NR
Littrup et al. [48]	2016	*Abdom Rad.*	RS	RFA	NR	99	6	20 **	2.79 **	LR 9.4%
Iitaka et al. [49]	2013	*Surg. Today*	CR	RFA	SCC	1	1	-	NR	OS 29 months LR 8 months
Blom et al. [50]	2013	*Ann. Surg. Onco.l*	RS	RT	Miscellaneous	493	117	15	NR	2-yr OS 20% LR NR
Tanaka et al. [51]	2010	*Dis. Esophagus.*	RS	RT	SCC	80	40	7	NR	2-yr OS 11.2% LR NR
Hong et al. [52]	2017	*J. Natl. Cancer Inst.*	PCT phase I/II	RT	Miscellaneous	89	12	30	2.5	3-yr LR 38.8% 3-yr OS 20.8% 3-yr DFS 9.2%
Rusthoven et al. [53]	2009	*J. Clin. Oncol.*	PCTPhase I/II	RT	NR	47	3	16	3	LR 0-8% 3-yr OS 10%
Fode et al. [54]	2015	*Radiother. Oncol.*	RS	RT	NR	321	2	60	3	2-yr LR 13% 5-yr OS 23%

OEC, oligometastatic esophageal cancer; RS, retrospective study; PCT, prospective clinical trial; OS, overall survival; DFS, disease-free survival; LR, local recurrence; NR, not reported; RFA, radiofrequency ablation; RT, radiotherapy. CR, case report. SCC, squamous cell carcinoma. * The values are expressed as the median. ** The value is expressed as the mean.
